# Assessment of Physical Activity Patterns in Adolescent Patients with Anorexia Nervosa and Their Effect on Weight Gain

**DOI:** 10.3390/jcm9030727

**Published:** 2020-03-07

**Authors:** Miriam Kemmer, Christoph U. Correll, Tobias Hofmann, Andreas Stengel, Julia Grosser, Verena Haas

**Affiliations:** 1 Department of Child and Adolescent Psychiatry, Charité-Universitätsmedizin Berlin, Corporate Member of Freie Universität Berlin, Humboldt-Universität zu Berlin, and Berlin Institute of Health, 13353 Berlin, Germany; miriam.kemmer@charite.de (M.K.); CCorrell@northwell.edu (C.U.C.); julia.grosser@charite.de (J.G.); 2 Donald and Barbara Zucker School of Medicine at Hofstra/Northwell, Hempstead, NY 11549, USA; 3 Department of Psychiatry, The Zucker Hillside Hospital, Glen Oaks, NY 11004, USA; 4 Center for Internal Medicine and Dermatology, Department for Psychosomatic Medicine, Charité-Universitätsmedizin Berlin, Corporate Member of Freie Universität Berlin, Humboldt-Universität zu Berlin, and Berlin Institute of Health Berlin, 12200 Berlin, Germany; tobias.hofmann@charite.de (T.H.); Andreas.Stengel@med.uni-tuebingen.de (A.S.); 5 Department of Psychosomatic Medicine and Psychotherapy, Medical University Hospital Tübingen, 72076 Tübingen, Germany

**Keywords:** anorexia nervosa, physical activity, accelerometry, weight gain

## Abstract

(1) Background: Altered physical activity (PA) affects weight recovery in anorexia nervosa (AN) patients. The study aimed to objectively characterize PA patterns and their effect on weight trajectory in adolescent AN patients. (2) Methods: PA was assessed in 47 patients on admission to inpatient treatment, in *n* = 25 of these patients again 4 weeks after discharge (follow-up, FU), as well as in 20 adolescent healthy controls using the Sense Wear™ armband. The following PA categories were defined by metabolic equivalent (MET) ranges: sedentary behavior (SB), light (LPA), moderate (MPA), vigorous (VPA), and high-level PA (HLPA= MPA + VPA). (3) Results: LPA on admission was significantly higher in AN patients than in controls (103 vs. 55 min/d, *p* < 0.001), and LPA in AN decreased over time to 90 min/d (*p* = 0.006). Patients with higher admission LPA (*n* = 12) still had elevated LPA at FU (*p* = 0.003). High admission LPA was associated with a higher inpatient BMI percentage gain (ΔBMI%; 18.2% ± 10.0% vs. 12.0% ± 9.7%, *p* = 0.037) but with a loss of ΔBMI% at FU (−2.3% ± 3.6% vs. 0.8% ± 3.6%, *p* = 0.045). HLPA at baseline was associated with a lower inpatient ΔBMI% (*p* = 0.045). (4) Conclusion: Elevated LPA in AN patients decreased after inpatient treatment, and PA patterns had an impact on weight trajectory.

## 1. Introduction

Anorexia nervosa (AN) is characterized by the restriction of energy intake, low body weight, fear of weight gain, and distorted body image [1]. Increased physical activity (PA) has been observed in 31–80% of patients suffering from AN [2], yet varying definitions and terminology, such as hyperactivity [3], excessive activity, and problematic exercise [4], are used in the literature to describe this phenomenon. These definitions include different types of PA, ranging from light PA, such as standing and walking, to high-level PA, such as running or biking. Due to the lack of a common definition, the AN-specific PA patterns as well as the effect of these PA patterns on the illness course are difficult to discern [4]. Additionally, when assessing PA in AN patients, there is a discrepancy between self-reported and objectively measured PA; patients tend to both either over- or underestimate their PA [5,6,7]. Despite the high clinical relevance of objectively measured PA in AN patients, considering its impact on weight recovery [8,9], few studies have objectively assessed PA in AN patients.

In the present study, the terms high-level PA and light PA will be used to distinguish between high and low intensity PA. In previous studies, average total PA did not vary between AN patients and healthy controls [5,10], but high-level PA was both higher [11] and lower [12] than in healthy controls. Several studies demonstrated increased light PA in AN patients, such as more time on feet during daytime [8], more time spent in PA intensities of between 1.8 and 3 metabolic equivalents (METs) [12], and more time ‘fidgeting’ compared to healthy controls [8,13]. However, varying definitions for fidgeting have been used, such as changes in body position while seated per time unit, and average acceleration from both feet in meters/second^2^/minute. There is high interpersonal variation in PA patterns among AN patients [11,12]. Longitudinal PA assessment indicated a link between pre-hospital exercise behavior and objectively measured total PA at the time of admission [14]. The findings on long-term patterns of the PA behavior of AN patients during and after treatment are controversial, as some studies state that total PA increased during weight restoration [8,15], while in others total PA decreased [16]. After discharge from inpatient treatment, both light PA and high-level PA remained constant [16], and, overall, PA did not vary between recovered AN patients and healthy controls [17]. Little data exist on how objectively measured PA affects weight trajectory. One study found no association between light PA (<3 METs) and BMI trajectory in adult AN patients [18], while others found that BMI trajectories were associated with time on feet when weight restored [8], the number of steps per day, the time spent in light PA (1.8–3.0 METs) [12], and the time spent in high-level PA (3–6 METs) [12,19]. In an outpatient setting, higher levels of total PA were associated with higher BMI values [20]. While this finding is somewhat counterintuitive, i.e., more PA that may have been driven by the desire to lose weight did not reduce BMI, it also highlights the bidirectional interdependence of BMI and PA, as it is also possible that a higher BMI reflects a healthier state that can result in higher and healthy activity.

Prior studies in an inpatient setting have shown that even under PA restricted inpatient conditions, there is a high variance in regards to PA amongst AN patients [11,12,16]. In one study, steps ranged from 2479 to 31,876 per day [12]. This variance is observable even when PA is specifically restricted as part of the treatment program [16]. Our study aims to better characterize these PA patterns and to assess their impact on weight trajectory in order to identify subgroups of patients at risk for a poorer treatment outcome. Identifying these patients at the beginning of treatment may allow for future studies to explore new approaches to treatment tailored to the needs of patients with specific types of hyperactivity.

We propose that PA levels impact weight trajectory, while not all physical activity levels will have the same impact on the weight recovery of patients. Based on our prior research [12], we hypothesize that high light physical activity will correlate with a poorer weight trajectory while increased high level physical activity will not have this same impact. We also propose that physical activity patterns are closely linked to the phenotype of AN; therefore, PA patterns will vary between adolescent AN patients and healthy controls. To pursue these aims, the following hypotheses were tested:

- Similar to adults, inpatient adolescent AN patients spend more time in light PA than age-matched healthy controls, while moderate PA and vigorous PA will be lower in AN patients.
- Within AN patients, different subgroups exist with respect to PA patterns (i.e., increased light PA or high-level PA defined as moderate PA + vigorous PA), and this PA pattern is an individual trait that remains constant over time, irrespective of therapy.
- More time spent in light PA, but not in high-level PA, on admission is a significant risk factor for lower inpatient weight gain and greater weight loss between discharge and outpatient follow-up.

## 2. Experimental Section

### 2.1. Study Populations

Adolescent female patients (age 12–18 years) hospitalized between 2014–2018 in the Department of Child and Adolescent Psychiatry, Psychosomatic Medicine and Psychotherapy at Charité-Universitätsmedizin Berlin were enrolled in this study. The inclusion criteria were: AN diagnosis (restricting, purging, and atypical subtype) according to International Statistical Classification of Diseases and Related Health Problems, 10th Revision (ICD-10). Patients diagnosed with a condition in addition to AN, which might significantly affect PA behavior (e.g., half-sided paralysis) were excluded. During the inpatient stay aimed at medical stabilization and weight recovery, all patients received psychotherapy, nutrition counselling, and body-oriented therapy. According to current German guidelines, target weight for discharge was set at the 25th BMI percentile, with an expected rate of weekly weight gain between 500 and 1000 g/week. PA was limited as part of the treatment program. Patients under the 3rd BMI percentile were given strict resting hours, one hour of sitting still after each mealtime and half an hour after each in-between meal. Resting times may have been prolonged based on individual treatment decisions. PA was limited to a 15-min walk a day and a one-hour yoga class per week focusing on relaxation techniques. Patients over the 3rd percentile were allowed to attend hospital school on an hourly basis. There was no mandatory bed rest, no one-on-one surveillance of patients, and patients were able to move freely in the ward. Patients over the 15th percentile were not given specific resting times and were able to attend a physical therapy group once a week. Additionally, patients who were clinically stable had the possibility to be granted a two-day leave. Patients were given dietary plans at the beginning of the program that they were instructed to adhere to, and mealtimes were supervised by clinical staff. On average, patients were given a plan of 1860 kilocalories (kcal) per day (range: 800–2600 kcal). The daily intake was increased by 200 kcal/ week in order to enable the targeted weight gain of 500g/week. Once patients achieved this weight gain, the meal plan was adjusted accordingly. In the patients participating in the study, no feeding tubes were used during the treatment. By the end of treatment program patients were free to make their own decisions about meals and did not have a specific dietary plan. Instead, patients were encouraged to stabilize their weight by making healthy choices about their food intake based on the nutritional training they had received during the treatment program. Information about illness duration, medication, admission weight and height, comorbidities, and length of stay was obtained from medical records.

We also recruited sex- and age-matched healthy controls between 2017–2018. The Sick, Control, One stone (14 lbs./6.5 kg.), Fat, Food (SCOFF) questionnaire was used in the screening process to identify and exclude all possible participants that exhibited early signs of altered eating behavior and/or negative body perception linked to body weight. Further exclusion criteria for healthy controls were any other physical or psychiatric diseases with a significant impact on PA behavior.

In total, 106 patients were approached about participating in the study, and 56 agreed to participate. Four patients were excluded retrospectively from analysis, because they were male, 5 datasets were excluded due to being incomplete. Forty-seven patient data sets were included in this analysis.

Forty-five possible participants for the control group responded to our informational online pamphlet. All of them received the screening questionnaire and 35 returned screening questionnaires to the study office. Of the healthy participants who completed the screening, 11 were excluded as part of the screening process. Twenty-five girls participated in the study assessment and in total 20 data sets were complete and included in the data analysis.

All participants and their legal guardians (if patients were <18 years old) provided written informed consent before participating in this study. The study was approved by the institutional ethics committee of the Charité-Universitätsmedizin Berlin (Identification code: EA2/034/14; date of approval 06/24/2014) and is in accordance with the Declaration of Helsinki on ‘Ethical Principles for Medical Research Involving Human Subjects’.

### 2.2. Anthropometry

Height and weight were measured in undergarments and empty-stomached during morning weigh-ins (7–8 a.m.) for patients at admission/discharge, and during the afternoon at follow-up, and similarly for healthy controls using a digital scale (KERN, MCB, Berlin, Germany) and a stadiometer (Sicca 2016, Hamburg, Germany).

### 2.3. Physical Activity Assessment

The SenseWear™ Pro3 Armband was used to assess PA. The SenseWear™ Pro3 is a two-axis accelerometer that also measures skin temperature, galvanic skin response and heat flux in order to calculate PA. It has been previously used in several studies to assess PA objectively both in controlled and free-range settings [21,22,23]. PA was assessed for three consecutive days, with recordings always taking place on Friday to Sunday, at the first study assessment and at outpatient follow-up. The same patients were given the SenseWear™ Pro3 within an average of 21 days of admission (first study assessment) and at the post- discharge outpatient follow-up visit, as described before [24], in order to assess longitudinal changes in activity; at both time points, patients were asked to wear the SenseWear™ Pro3 device on their dominant arm continuously for three consecutive days (data admissible if worn >20, 5 h on at least two out of the three days), except when showering, bathing, or swimming. As part of the inpatient treatment program, PA was limited; meanwhile, during post-discharge follow-up, PA was unrestricted. Healthy controls were given the SenseWear™ Pro3 Armband on one occasion on the day of their assessment and were instructed to wear the device for three consecutive days (Friday to Sunday) continuously except when showering, bathing, or swimming. PA was unrestricted in the control group.

In accordance with previous work [12,19], we defined PA intensity levels as follows:

- Sedentary behavior: ≥ 1.1 to ≤ 1.8 METs
- Light-intensity PA: > 1.8 and < 3 METs
- Moderate-intensity PA: ≥ 3 to < 6 METs
- Vigorous-intensity PA: ≥ 6 METs

For the purpose of this paper, the category very light PA used previously was renamed as sedentary behavior, as new research suggests that 1.1–1.8 METs are more in line with this terminology [25]. The following activities are associated with each category. Sedentary behavior (SB) includes behavior such as lying down, watching television, eating, sitting, reading, and standing. LPA includes light physical work, walking slowly (less than 2.0 miles per hour), household errands and activities of daily life such as getting ready for bed. MPA involves activities such as descending stairs, walking for pleasure, dance practice, low impact aerobics, and bicycling (less than 10 miles per hour). Vigorous PA (VPA) includes running (<15 min/mile), competitive football or dance and high intensity cycling [26].

### 2.4. Statistical Analysis

A *p*-value of 0.05 was set as the significance threshold. All variables were tested two-sided. Analyses were conducted using R version 3.5.3 (2019-03-11). Comparing high vs. low physical activity was limited to AN patients and was calculated via median split. Descriptive statistics were selected according to scale level as absolute and relative frequencies for categories, median, the 25th/75th percentile, and extreme values for ordinal data, and the mean, standard deviation, and extreme values for normally distributed continuous measures. Group comparisons were performed using Fisher’s exact test, the Wilcoxon rank sum test, and a t-test, accordingly. Correlations between measures were computed using the Spearman rank correlation. Range-based variability was calculated using the Siegel-Tukey test for equality in variability with adjustments for the median.

## 3. Results

### 3.1. Characterization of the Study Population

Within the study population of 47 patients, 25 patients (53%) were diagnosed with restrictive, 11 (23%) with purging, and 11 (23%) with atypical AN. Twenty-eight patients (60%) had their first inpatient admission, and, for the remaining patients, the number of prior inpatient therapies varied from 1 to 4. The mean illness duration was 11 months, ranging from 6.2–16.8 months. Thirty patients (64%) had comorbidities, including major depression (*n* = 9; 19%), borderline personality disorder (*n* = 3; 6%), anxiety disorders (*n* = 5; 11%), and obsessive-compulsive disorder (*n* = 5; 11%). Only eight patients (17%) received psychopharmacological medications, i.e., stimulating antidepressants (*n* = 4; 9%) and antipsychotics (*n* = 2; 4%). Three patients received oral contraceptives (6%). None of the healthy controls took any psychopharmacological medication, and 4 participants (20%) took oral contraceptives. The time between admission and first study assessment was on average 21 (2–50) days, and during this time, the patients’ weight had increased by 0.9 ± 1.1 (−2.6–3.5) kilogram (kg). Table 1 shows the clinical characteristics and PA parameters of the AN patients on admission compared to healthy controls. The number of steps was significantly lower in AN patients (*p* = 0.048), but there was no between-group difference in the range of steps. AN patients spent significantly more time in light PA (*p* < 0.001) and less in moderate PA (*p* = 0.009) than healthy controls.

### 3.2. General Parameters and Physical Activity of AN Patients and Healthy Controls

The median length of stay for all patients was 17.0 weeks (range: 9.0 to 28.4 weeks), with a weight change from admission to discharge from 42.2 ± 6.0 to 48.1 ± 4.8 kg (total weight gain: 5.96 ± 3.62 kg; rate of weight gain: 391 ± 245 g/week). Of the 47 patients, 25 (53%) returned for outpatient follow-up, 36 days (range: 27–119 days) after discharge. This subgroup had a body weight increase from 41.9 ± 4.4 kg to 48.9 ± 3.1 kg during hospitalization that lasted 17.0 (9.0 to 28.4) weeks, which translates into an increase of 6.98 ± 3.45 kg and a rate of weight gain of 445 ± 241 g/week. Compared to the patients who returned, the patients not returning for a follow-up visit had similar admission BMI and PA parameters, but were significantly younger (*p* = 0.006). On average, weight between discharge and follow-up remained constant at 48.3 ± 3.8 kg (*p* = 0.102; range: −3.8 to +3.4). However, at follow-up, body weight and BMI of the AN patients remained significantly lower than in healthy controls (*p* < 0.001).

Clinical characteristics and PA of the patient subgroup returning for their follow-up in comparison with healthy controls are shown in Table 2. At the first study assessment, AN patients had significantly lower body weight (*p* < 0.001), BMI (*p* < 0.001), and BMI percentile (*p* < 0.001), and spent significantly more time in light PA than healthy controls (*p* < 0.001). At outpatient follow-up, AN patients were significantly older (*p* < 0.001), had a significantly lower body weight (*p* < 0.001), BMI (*p* < 0.001), BMI percentile (*p* < 0.001), and spent more time in light PA (*p* = 0.039) and vigorous PA (*p* = 0.006) than healthy controls. From admission to follow-up, AN patients gained significant weight (*p* < 0.001), BMI (*p* < 0.001) and BMI percentile (*p* < 0.001), had higher number of daily steps (*p* = 0.037), and spent significantly less time in light PA (*p* = 0.008) and more time in vigorous PA (*p* < 0.001).

### 3.3. Light PA in AN Patients Over Time

**Figure 1:**
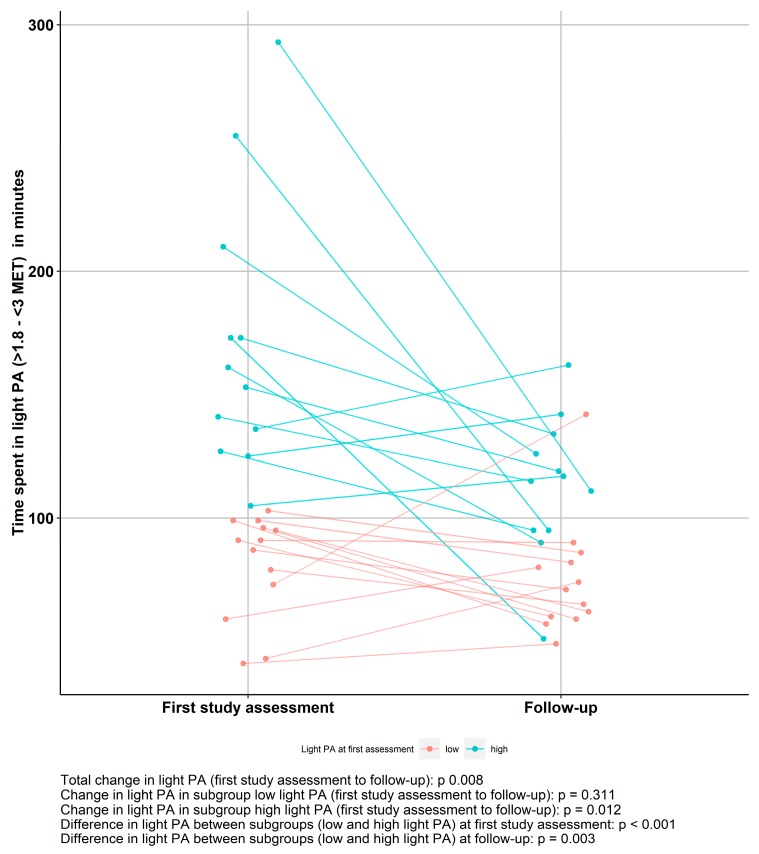
Light physical activity (PA) patterns in anorexia nervosa (AN) patients over time grouped by median split based on light PA at first study assessment (low light PA, *n* = 13; high light PA, *n* = 12).

As shown in Figure 1, total time spent in light PA in AN patients decreased significantly between the first study assessment and follow-up (*p* = 0.008). Nevertheless, at follow-up a significant difference between low light PA and high light PA patients remained, as high light PA patients continued to show higher levels of light PA at follow-up (*p* = 0.003). When analyzing the subgroups separately, the decrease in light PA over time was only significant in the group with high baseline light PA (*n* = 12, *p* < 0.001), and not in the low light PA group (*n* = 13, *p* = 0.147).

### 3.4. Impact of light PA/ High-Level PA at Admission on Weight Trajectory

Compared to patients with low baseline light PA (*n* = 13), those with high baseline light PA (*n* = 12) showed a significantly higher inpatient BMI percentage increase, but less significant outpatient BMI percentage improvement (Table 3). Although the average time spent in high-level PA was short, increased high-level PA had a negative impact on inpatient BMI percentage change, as patients with higher baseline levels of high-level PA (*n* = 11) had significantly lower inpatient BMI percentage change than those with lower baseline levels of high-level PA (*n* = 14). Nevertheless, there were no differences in BMI percentage change in the outpatient setting (Table 3).

### 3.5. Characteristics of Patients Grouped by Low/High Levels of Light PA and Longitudinal Impact of Time Spent in Light PA

Comparing the two subgroups of patients (low light PA, *n* = 23; high light PA, *n* = 24) at admission, there were no differences with regards to AN subtype, comorbidities, medication, the presence of amenorrhea, hormonal contraception, age, and duration of illness (Ref. Table A1). The two subgroups presented a similar duration of inpatient stay and height. However, patients with high levels of light PA did have a significantly lower weight (*p* = 0.015), BMI (*p* < 0.001), and BMI percentile (*p* = 0.026) than low light PA patients. This difference was still present at discharge, where high light PA AN patients continued to present a significantly lower weight (*p* < 0.001), BMI (*p* < 0.001), and BMI percentile (*p* = 0.018). There was no significant difference in the BMI change between the first study assessment and discharge, or in daily steps. High light PA AN patients spent less time in sedentary behavior than low light PA AN patients (*p* < 0.001), but had similar levels of moderate PA and vigorous PA.

At outpatient follow-up, there was no significant difference with regards to age and height between the two subgroups. High light PA AN had significantly lower weight (*p* = 0.003) and BMI percentile (*p* = 0.042); but there was no significant difference in the BMI. High light PA AN patients had a lower BMI percentage change between discharge and outpatient follow-up (*p* = 0.045). There was no significant difference in daily steps and sedentary behavior. High light PA AN patients continued to present significantly higher light PA values (*p* = 0.003). Moderate PA was comparable and high light PA AN patients presented lower amounts of vigorous PA (*p* = 0.034).

**Figure 2:**
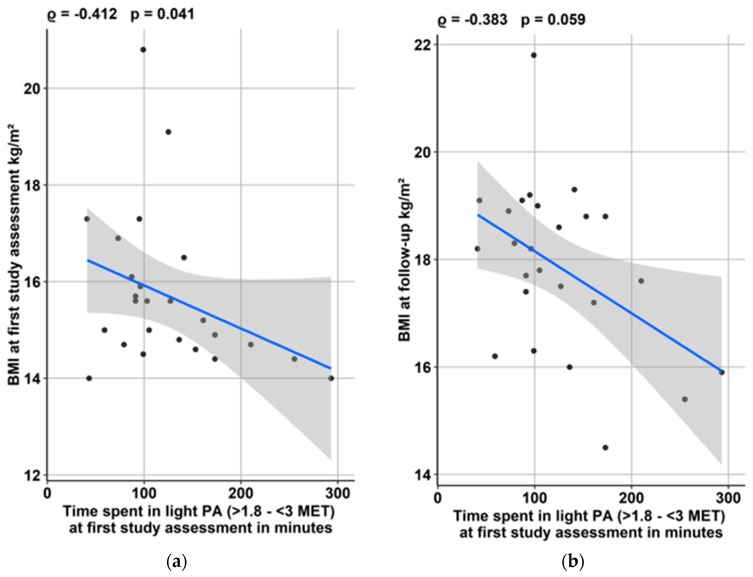
Impact of time spent in light physical activity (PA) at first study assessment (**a**) on admission BMI and (**b**) BMI at outpatient follow-up.

The time spent in light PA at first study assessment showed a significant association with BMI at admission (*p* = 0.041), while time spent in light PA at admission and the BMI at outpatient follow-up were only associated trend-level significance (*p* = 0.059) (Figure 2).

## 4. Discussion

Objectively assessing PA patterns in adolescent AN patients compared to healthy controls and their impact on the weight trajectories of AN patients yielded following results: (1) There was no difference in sedentary behavior, AN inpatients exhibited more light PA, less moderate PA, and similar vigorous PA compared to healthy controls; (2) The time spent in light PA by AN patients decreased between admission and outpatient follow-up, but patients who had spent relatively more time in light PA on admission continued to do so at outpatient follow-up; (3) The decrease in light PA over time was only significant in the subgroup with high baseline light PA; (4) Contrary to our hypothesis, high baseline light PA was associated with a higher inpatient BMI percentage change, but as expected, with a poorer outpatient BMI percentage change; (5) High-level PA had a negative impact on inpatient but not on outpatient BMI percentage change.

### 4.1. Comparison of Activity Patterns Between AN Patients and Healthy Controls

Consistent with our previous study in adult AN inpatients [19], adolescent AN patients in the present study spent significantly more time in light PA than healthy, age-matched controls. High light PA was not mirrored by a high daily step count, pointing out the importance of a detailed PA assessment using intensity categories. In the literature, “fidgeting” assessed using a shoe-based monitor was higher in AN inpatients [13] than in controls and was similar when assessed using the Intelligent Device for Energy Expenditure and Activity (IDEEA™) accelerometer [8]. A comparison of data is difficult due to inconsistent definitions of light PA and different PA dimensions being assessed with varying devices. ‘Fidgeting’ in the second study may be more similar to sedentary behavior in the present study, which was similar between the two groups. For high-level PA, controversial results have been reported. In the present study, AN patients spent less time in moderate PA, and similar time in vigorous PA. A previous study using the Actiwatch™ (AW7) in a day hospital setting found similar amounts of high-level PA in AN and control subjects (no distinction was made between moderate PA and vigorous PA) [27], while a study using the SenseWear™ Armband in inpatient adolescent and adult AN patients reported higher high-level PA than in controls (3–6 METs, which corresponds to the category of moderate PA in the present study) [11]. The different amounts of high-level PA may be a result of different treatment programs and varying approaches to PA restriction. No information about the handling of PA during treatment was given in the cited studies. Furthermore, a variation in PA behavior patterns within the different control groups and different recruitment locations may have impacted the results.

### 4.2. Longitudinal Development of Light PA in AN Patients

The current study assessed light PA longitudinally at the beginning of inpatient treatment and at outpatient follow-up. While on average, light PA decreased over time, there were two characteristic subgroups: patients grouped in either high or low light PA at the first study assessment continued to exhibit these same PA patterns at follow-up. These findings lead to the question as to whether time spent in high or low light PA is an individual trait, which persists over time, regardless of therapy and setting, or whether the time needed to normalize varies and takes up more than a median of 36.0 days. Casper et al. proposed a dysregulation of PA called ‘restless activation’ as a phenotype of AN and hypothesized that it may be linked to improved self-esteem and wellbeing [28]. In rodent models, PA has been linked to dopamine and endocannabinoid signaling networks that suggest an addictive property [29]. Similar findings using MRI imagining in AN patients have linked altered neurological responses in the reward system to excessive exercise [30]. We are only aware of one comparable study, which reported a trend for increased time spent on feet between low-weight and weight restored AN patients, while ‘fidgeting’ did not vary when using the IDEEA™ [8]. The varying age of the study populations (adolescent and adult patients vs. only adolescents in the present study), the different admission BMIs (16.1 ± 1.0 vs. 15.6 ± 1.8 in the current study), and the different measurement instruments may have impacted PA behaviors and readings.

### 4.3. Impact of PA Patterns on Weight Trajectory

Contrary to our hypothesis, increased time spent in light PA was linked to a higher inpatient BMI percentage change. Previous studies on the impact of light PA on inpatient weight trajectory have yielded mixed findings. In an exploratory, non-linear model based on 50 adult AN patients, time spent in light PA was a potential predictor for poor BMI increase during inpatient treatment [19]. In adolescent and adult AN patients, time spent in light PA was inversely linked to BMI at discharge, while there was no significant relationship to BMI percentage change [19]. No association between ‘fidgeting’, assessed with a shoe-based accelerometer, and weight gain was found in a group of 11 adolescent and adult AN patients [13]. Age differences may have led to different results, as in healthy participants, PA behaviors vary between adolescents and adults. Healthy adolescents are more active and spent more time in high-level PA than adults [31,32]. Additionally, some of these previous studies were small. In the present study, the group of patients with high light PA had a significantly lower BMI at admission; a higher BMI increase in this group may have been caused the program’s discharge weight target at the 25th BMI percentile requiring more weight gain in this group in order to be discharged. There also might be a link between increased LPA and muscle gain as it has been shown that LPA can improve muscle strength [33], which may contribute to the increased BMI percentage change within the high LPA group. The present study found a statistical trend (*p* = 0.059) for an inverse association between high light PA at admission and inpatient BMI gain, as well as a significant inverse association with BMI percentile at follow-up. Therefore, the assessment of PA patterns at admission might hold the potential to identify patients at risk of poor weight gain. The time spent on feet in 61 AN patients at the assessment point weight, but not at low weight, was linked to a poorer weight trajectory at the 12 month follow-up [8]. Heterogeneous results may thus be caused either by different time points of PA assessment during treatment or by different follow-up durations. Consistent with our previous study [19], high-level PA was inversely associated with inpatient BMI change. Conversely, in a group of 88 AN patients in a day hospital setting, high-level PA assessed using an Actiwatch™ (AW7) was directly associated with achieving BMI > 18.5 and with decreased AN-specific cognitions and reduced binge/purge behavior [27]. Different settings (inpatient vs. day hospital treatment) may have impacted the patients’ ability to exercise at higher intensities and the impact high-level PA had on the weight trajectory.

When assessing the impact of PA patterns on weigh status in healthy adolescents, two trends can be observed: high levels of high-level PA (HLPA) are associated with a lower BMI and a lower body fat mass. Additionally, more time in sedentary behavior is linked to a higher BMI and higher body fat [34,35,36]. These trends are also replicable when analyzing only healthy female participants [37,38]. Further sedentary behavior in both gender-mixed groups and female-only groups was also linked to a higher BMI increase in the long run [39,40]. However, in a sample of female secondary school students, there was no correlation between being underweight and PA patterns (both SB and HLPA) [41].

### 4.4. Limitations

The limitations of this study first include the prolonged and variable times between the admission and the first study assessment, and between discharge and the follow-up assessment. Second, a high drop-out (*n* = 22) may have reduced the generalizability of the findings, yet comparable studies had both similar (*n* = 26) [8] and lower (*n* = 3) [16] drop-outs. Third, there was a significant age difference between the patients at follow-up and the healthy controls. Fourth, the validity of the SenseWear™ Pro 3 Armand has not been assessed for PA parameters in underweight AN patients; thus, the validity of the obtained data is unknown. Fifth different cutoff points for PA are used in the literature. We used a definition for sedentary behavior (very low PA) with a cutoff point at 1.8 METs [42] to establish compatibility with our previous work [12,19], but a new definition proposed a cutoff point at 1.5 METs [25]. Therefore, the actual activities associated with sedentary behavior in this study may not fully represent all aspects of sedentary behavior. Sixth, it is unknown if wearing a SenseWear™ Armand affects PA behavior. Seventh, no data about the caloric intake of patients after discharge were obtained. Finally, potential differences in PA based on socioeconomic status were not assessed. However, despite these limitations, this is one of the first studies to assess the longitudinal development of light PA and its impact on weight trajectories in adolescent AN patients.

## 5. Conclusions

In summary, PA patterns vary between AN patients and healthy controls and impact weight trajectories in AN patients. Our study aims to raise awareness of the different types of hyperactivity and their impact on the weight development of AN patients. Our research may provide a foundation for future research into the benefit of objectively measured PA patterns in AN patients and may allow for a better understanding of PA as a disease maintaining factor as well as the identification of high risk patients at the beginning of treatment. This could enable research into new treatment interventions that are tailored to the different PA subgroups of patients, such as high light PA patients. An early intervention that focuses on reducing light PA hyperactivity from the beginning of the treatment program on may positively impact short- and long-term weight development. Our study shows that the detailed assessment of PA patterns, rather than general PA parameters, such as steps or activity counts, provides valuable insight into PA behavior of AN patients.

## Figures and Tables

**Table 1:** General characteristics and physical activity parameters of patients with AN at first study assessment compared to healthy controls.

Participant Characteristics and PA	AN Total (*n* = 47)	Healthy Controls (*n* = 20)	*p*-Value
Age, years	15.70 (14.68/16.64) (12.44–17.85)	15.02 (13.59/15.84) (12.07–17.86)	0.139
Height, centimeters	164.3 ± 6.4 (150.2–183.0)	165.1 ± 7.1 (153.0–178.0)	0.672
Weight, kilograms	42.2 ± 6.0 (31.3–58.2)	57.3 ± 9.4 (38.8–70.8)	<0.001
BMI, kilogram/meter^2^	15.60 ± 1.78 (12.80–20.40)	20.96 ± 2.88 (16.00–27.60)	<0.001
BMI, percentile	4.6 ± 9.9 (0.0–43.0)	51.8 ± 25.5 (7.0–93.0)	<0.001
Steps	8430 (6522/10398) (2026–26439)	11390 (8261/13680) (4427–23139)	0.048
Sedentary behavior (min) (≥1.1 to ≤1.8 METs)	705 (624/765) (189–868)	647 (557/732) (386–853)	0.118
Light PA (min) (>1.8 and <3 METs)	105 (73/204) (41–530)	55 (42/88) (14–303)	<0.001
Moderate PA (min) (≥3 to <6 METs)	77.0 (44.3/114.2) (3.0–268.0)	121.5 (82.3/188.2) (25.0–302.0)	0.009
Vigorous PA (min) (≥6 METs)	2.0 (0.0/10.2) (0.0–212.0)	2.0 (0.4/15.8) (0.0–55.0)	0.697

Values are means ± SDs (range) (first quartile/ third quartile). AN, anorexia nervosa; BMI, body mass index; MET, metabolic equivalent of task; PA, physical activity.

**Table 2:** General characteristics and PA parameters for AN patients at first study assessment, AN patients at outpatient follow-up and healthy controls °.

Participant Characteristics and PA	AN (First Study Assessment) (*n* = 25)	AN (Follow-Up) (*n* = 25)	Healthy Controls (*n* = 20)
Age, years	16.49 (15.08/17.38) (12.44–17.85)	16.90 (15.49/17.82) ^b^ (12.88–18.20)	15.02 (13.59/15.84) ^2^ (12.07–17.86)
Height, centimeters	165.4 ± 5.5 (156.3–183.0)	164.9 ± 5.1 ^a^ (156.6–181.3)	165.1 ± 7.1 (153.0–178.0)
Weight, kilograms	41.9 ± 4.4 (35.3– 53.0)	48.3 ± 3.8 ^b^ (41.1–56.6)	57.3 ± 9.4 ^II 2^ (38.8–70.8)
BMI, kilogram/meter^2^	15.34 ±1.72 (12.80–20.40)	17.87 ± 1.56 ^b^ (14.50–21.80)	20.96 ± 2.88 ^II 2^ (16.00–27.60)
BMI, percentile	2.8 ± 8.9 (0.0–43.0)	13.2 ± 13.7 ^b^ (0.0–61.0)	51.8 ± 25.5 ^II 2^ (7.0–93.0)
Steps	7821 (5948/9296) (2753–23923)	10475 (8612/14975) ^a^ (646–23273)	11390 (8261/13680) (4427–23139)
Sedentary behavior (min) (≥1.1 to ≤ 1.8 METs)	712 (634/760) (483–868)	681 (636/747) (499–848)	647 (557/732) (386–853)
Light PA (min) (>1.8 and < 3 METs)	103.0 (89.7/155.7) (41.0–293.0)	90.0 (64.0/117.7) ^a^ (49.0–162.0)	55.0 (41.8/88.1) ^II 2^ (14.0–303.0)
Moderate PA (min) (≥3 to < 6 METs)	73.0 (35.7/108.7) (22.0–268.0)	96.0 (73.0/132.3) (3.0–275.0)	121.5 (82.3/188.2) (25.0–302.0)
Vigorous PA (min) (≥6 METs)	1.0 (0.0/3.3) (0.0–23.0)	13.0 (5.0/33.3) ^b^ (0.0–116.0)	2.0 (0.4/15.8) ^1^ (0.0–55.0)

° The values are means ± SDs (range) (first quartile/ third quartile). AN, anorexia nervosa; BMI, body mass index; MET, metabolic equivalent of task; PA, physical activity; AN (first study assessment) vs. AN (follow-up): ^a^, *p* < 0.05; ^b^, *p* < 0.01; AN (first study assessment) vs. healthy controls: ^II^, *p* < 0.01; AN (follow-up) vs. healthy controls: ^1^, *p* < 0.05; ^2^, *p* < 0.01.

**Table 3:** Impact of light PA and high-level PA in anorexia nervosa patients at admission on BMI trajectory (BMI percentage change) from admission to discharge and from discharge to outpatient follow-up.

Weight Trajectory in PA Subgroups	Low Light PA (*n* = 13)	High Light PA (*n* = 12)	*p*-value	Low HLPA (*n* = 14)	High HLPA (*n* = 11)	*p*-value
Inpatient BMI percentage change (admission to discharge)	12.0 ± 9.7 (−3.6–33.6)	18.2 ± 10.0 (−0.5–38.6)	0.037	21.3 ± 9.7 (7.7–38.6)	13.43 ± 8.50 (−0.52–25.83)	0.045
Outpatient BMI percentage change (discharge to follow-up)	0.80 ± 3.61 (−3.70–9.42)	−2.28 ± 3.63 (−7.59–4.49)	0.045	−0.91 ± 3.90 (−6.25–9.42)	−0.38 ± 4.02 (−7.59–5.96)	0.740

Values are means ± SDs (range) (first quartile/ third quartile); unit = %; BMI, body mass index; HLPA, high level physical activity; PA, physical activity.

## Appendix A

**Table A1:** General characteristics and PA parameters of AN patients at admission, first study assessment and discharge grouped by a low/high level of light PA (median split).

Participant Characteristics and PA	Low Light PA (*n* = 23)	High Light PA (*n* = 24)	*p*-value
Duration of illness, months	12.0 (7.3/15.8) (3.0–64.0)	9.0 (4.0/17.6) (1.0–56.0)	0.176
Admission age, y	15.82 (14.87/16.64) (13.78–17.85)	15.11 (13.89/16.70) (12.44–17.82)	0.250
Admission height, cm	166.7 ± 6.4 (159.0–183.0)	164.1 ± 4.3 (156.3–170.0)	0.253
Admission weight, kg	43.9 ± 4.1 (39.6–53.0)	39.8 ± 3.8 (35.3–46.5)	0.015
Admission BMI, kg/m^2^	15.84 ± 1.73 (13.90–20.40)	14.80 ± 1.61 (12.80–19.10)	0.135
Admission BMI percentile (%)	4.1 ± 11.7 (0.0–43.0)	1.33 ± 4.31 (0.00–15.00)	0.443
Steps	8586 (7136/9735) (2753–24536)	7870 (6159/12179) (2026–26439)	0.882
Sedentary behavior (min) (≥1.1 to ≤1.8 METs)	730 (698/789) (581–856)	646 (497/715) (189–868)	<0.001
Light PA (min) (>1.8 and <3 METs)	73.0 (54.3/93.5) (41.0–103.0)	192 (146/273) (105–530)	<0.001
Moderate PA (min) (≥3 to <6 METs)	73.0 (46.5/103.5) (22.0–268.0)	89.5 (39.3/120.8) (3.0–241.0)	0.663
Vigorous PA (min) (≥6 METs)	2.0 (1.0/10.0) (0.0–54.0)	1.5 (0.0/8.9) (0.0–212.0)	0.729
Time between admission and discharge, days	95.0 (74.8/118.8) (47.0–225.0)	121.0 (88.8/135.6) (41.0–171.0)	0.097
Discharge weight, kg	51.4 ± 3.3 (45.3–61.1)	45.0 ± 3.9 (36.6–52.5)	<0.001
Discharge BMI, kg/m^2^	18.38 ± 0.96 (16.50–20.50)	17.31 ± 1.15 (15.50–19.40)	<0.001
Discharge BMI percentile (%)	18.4 ± 11.1 (0.0–45.0)	11.4 ± 8.4 (0.0–36.0)	0.018
BMI change from first study assessment to discharge kg/m²	1.85 ± 1.41 (−0.70–4.70)	2.60 ± 1.36 (−0.10–5.40)	0.072
BMI percentile change from first study assessment to discharge (%)	12.0 ± 9.7 (−3.6–33.6)	18.2 ± 10.0 (−0.5–38.6)	0.037

Values are means ± SDs (range) (first quartile/ third quartile). AN, anorexia nervosa; BMI, body mass index; METs, metabolic equivalents; PA, physical activity.

## References

[1] Association, American Psychiatric (2013). Diagnostic and Statistical Manual of Mental Disorders: Dsm-5.

[2] Solenberger S.E. (2001). Exercise and eating disorders: A 3-year inpatient hospital record analysis. Eat. Behav..

[3] Achamrah N., Coeffier M., Dechelotte P. (2016). Physical activity in patients with anorexia nervosa. Nutr. Rev..

[4] Rizk M., Lalanne C., Berthoz S., Kern L., Godart N. (2015). Problematic Exercise in Anorexia Nervosa: Testing Potential Risk Factors against Different Definitions. PLoS ONE.

[5] Keyes A., Woerwag-Mehta S., Bartholdy S., Koskina A., Middleton B., Connan F., Webster P., Schmidt U., Campbell I.C. (2015). Physical activity and the drive to exercise in anorexia nervosa. Int. J. Eat. Disord..

[6] Alberti M., Galvani C., Capelli C., Lanza M., El Ghoch M., Calugi S., Dalle Grave R. (2013). Physical fitness before and after weight restoration in anorexia nervosa. J. Sports Med. Phys. Fit..

[7] Bratland-Sanda S., Sundgot-Borgen J., Ro O., Rosenvinge J.H., Hoffart A., Martinsen E.W. (2010). “I’m not physically active - I only go for walks”: Physical activity in patients with longstanding eating disorders. Int. J. Eat. Disord..

[8] Gianini L.M., Klein D.A., Call C., Walsh B.T., Wang Y., Wu P., Attia E. (2016). Physical activity and post-treatment weight trajectory in anorexia nervosa. Int. J. Eat. Disord..

[9] Gummer R., Giel K.E., Schag K., Resmark G., Junne F.P., Becker S., Zipfel S., Teufel M. (2015). High Levels of Physical Activity in Anorexia Nervosa: A Systematic Review. Eur. Eat. Disord. Rev..

[10] Hechler T., Rieger E., Touyz S., Beumont P., Plasqui G., Westerterp K. (2008). Physical activity and body composition in outpatients recovering from anorexia nervosa and healthy controls. Adapt. Phys. Act. Q..

[11] El Ghoch M., Calugi S., Pellegrini M., Milanese C., Busacchi M., Battistini N.C., Bernabe J., Dalle Grave R. (2013). Measured physical activity in anorexia nervosa: Features and treatment outcome. Int. J. Eat. Disord..

[12] Lehmann C.S., Hofmann T., Elbelt U., Rose M., Correll C.U., Stengel A., Haas V. (2018). The Role of Objectively Measured, Altered Physical Activity Patterns for Body Mass Index Change during Inpatient Treatment in Female Patients with Anorexia Nervosa. J. Clin. Med..

[13] Belak L., Gianini L., Klein D.A., Sazonov E., Keegan K., Neustadt E., Walsh B.T., Attia E. (2017). Measurement of fidgeting in patients with anorexia nervosa using a novel shoe-based monitor. Eat. Behav..

[14] Klein D.A., Mayer L.E., Schebendach J.E., Walsh B.T. (2007). Physical activity and cortisol in anorexia nervosa. Psychoneuroendocrinology.

[15] Bratland-Sanda S., Sundgot-Borgen J., Ro O., Rosenvinge J.H., Hoffart A., Martinsen E.W. (2010). Physical activity and exercise dependence during inpatient treatment of longstanding eating disorders: An exploratory study of excessive and non-excessive exercisers. Int. J. Eat. Disord..

[16] Kostrzewa E., van Elburg A.A., Sanders N., Sternheim L., Adan R.A., Kas M.J. (2013). Longitudinal changes in the physical activity of adolescents with anorexia nervosa and their influence on body composition and leptin serum levels after recovery. PLoS ONE.

[17] Dellava J.E., Hamer R.M., Kanodia A., Reyes-Rodriguez M.L., Bulik C.M. (2011). Diet and physical activity in women recovered from anorexia nervosa: A pilot study. Int. J. Eat. Disord..

[18] El Ghoch M., Calugi S., Pellegrini M., Chignola E., Dalle Grave R. (2016). Physical activity, body weight, and resumption of menses in anorexia nervosa. Psychiatry Res..

[19] Großer J., Hofmann T., Stengel A., Zeeck A., Winter S., Correll C.H. (2018). Psychological and Nutritional Correlates of Objectively Assessed Physical Activity in Patients with Anorexia Nervosa, Submitted.

[20] Bouten C.V., van Marken Lichtenbelt W.D., Westerterp K.R. (1996). Body mass index and daily physical activity in anorexia nervosa. Med. Sci. Sports Exerc..

[21] Calabro M.A., Lee J.M., Saint-Maurice P.F., Yoo H., Welk G.J. (2014). Validity of physical activity monitors for assessing lower intensity activity in adults. Int. J. Behav. Nutr. Phys. Act..

[22] van Hoye K., Mortelmans P., Lefevre J. (2014). Validation of the SenseWear Pro3 Armband using an incremental exercise test. J Strength Cond. Res..

[23] Johannsen D.L., Calabro M.A., Stewart J., Franke W., Rood J.C., Welk G.J. (2010). Accuracy of armband monitors for measuring daily energy expenditure in healthy adults. Med. Sci. Sports Exerc..

[24] Stengel A., Haas V., Elbelt U., Correll C.U., Rose M., Hofmann T. (2017). Leptin and Physical Activity in Adult Patients with Anorexia Nervosa: Failure to Demonstrate a Simple Linear Association. Nutrients.

[25] Tremblay M.S., Aubert S., Barnes J.D., Saunders T.J., Carson V., Latimer-Cheung A.E., Chastin S.F.M., Altenburg T.M., Chinapaw M.J.M. (2017). Sedentary Behavior Research Network (SBRN)—Terminology Consensus Project process and outcome. Int. J. Behav. Nutr. Phys. Act..

[26] Ainsworth B.E., Haskell W.L., Herrmann S.D., Meckes N., Bassett D.R.J., Tudor-Locke C., Greer J.L., Vezina J., Whitt-Glover M.C., Leon A.S. (2011). 2011 Compendium of Physical Activities: A second update of codes and MET values. Med. Sci. Sports Exerc..

[27] Sauchelli S., Arcelus J., Sanchez I., Riesco N., Jimenez-Murcia S., Granero R., Gunnard K., Banos R., Botella C., de la Torre R. (2015). Physical activity in anorexia nervosa: How relevant is it to therapy response?. Eur. Psychiatry J. Assoc. Eur. Psychiatr..

[28] Casper R.C. (2018). Not the Function of Eating, but Spontaneous Activity and Energy Expenditure, Reflected in “Restlessness” and a “Drive for Activity” Appear to Be Dysregulated in Anorexia Nervosa: Treatment Implications. Front. Psychol..

[29] Garland T.J., Schutz H., Chappell M.A., Keeney B.K., Meek T.H., Copes L.E., Acosta W., Drenowatz C., Maciel R.C., van Dijk G. (2011). The biological control of voluntary exercise, spontaneous physical activity and daily energy expenditure in relation to obesity: Human and rodent perspectives. J. Exp. Biol..

[30] Kullmann S., Giel K.E., Hu X., Bischoff S.C., Teufel M., Thiel A., Zipfel S., Preissl H. (2014). Impaired inhibitory control in anorexia nervosa elicited by physical activity stimuli. Soc. Cogn. Affect Neurosci..

[31] Gordon-Larsen P., Nelson M.C., Popkin B.M. (2004). Longitudinal physical activity and sedentary behavior trends: Adolescence to adulthood. Am. J. Prev. Med..

[32] Nelson M.C., Gordon-Larsen P., Adair L.S., Popkin B.M. (2005). Adolescent physical activity and sedentary behavior: Patterning and long-term maintenance. Am. J. Prev. Med..

[33] Chantler I., Szabo C.P., Green K. (2006). Muscular strength changes in hospitalized anorexic patients after an eight week resistance training program. Int. J. Sports Med..

[34] Werneck A.O., Silva E.C.A., Bueno M.R.O., Vignadelli L.Z., Oyeyemi A.L., Romanzini C.L.P., Ronque E.R.V., Romanzini M. (2019). Association(s) Between Objectively Measured Sedentary Behavior Patterns and Obesity Among Brazilian Adolescents. Pediatric Exerc. Sci..

[35] Augustin N.H., Mattocks C., Cooper A.R., Ness A.R., Faraway J.J. (2012). Modelling fat mass as a function of weekly physical activity profiles measured by actigraph accelerometers. Physiol. Meas..

[36] Machado-Rodrigues A.M., Coelho-e-Silva M.J., Mota J., Padez C., Ronque E., Cumming S.P., Malina R.M. (2012). Cardiorespiratory fitness, weight status and objectively measured sedentary behaviour and physical activity in rural and urban Portuguese adolescents. J. Child Health Care Prof. Work. Child. Hosp. Community.

[37] Allender S., Kremer P., de Silva-Sanigorski A., Lacy K., Millar L., Mathews L., Malakellis M., Swinburn B. (2011). Associations between activity-related behaviours and standardized BMI among Australian adolescents. J. Sci. Med. Sport.

[38] Jones M.A., Skidmore P.M., Stoner L., Harrex H., Saeedi P., Black K., Barone Gibbs B. (2020). Associations of accelerometer-measured sedentary time, sedentary bouts, and physical activity with adiposity and fitness in children. J. Sports Sci..

[39] Altenburg T.M., Singh A.S., van Mechelen W., Brug J., Chinapaw M.J. (2012). Direction of the association between body fatness and self-reported screen time in Dutch adolescents. Int. J. Behav. Nutr. Phys. Act..

[40] Hands B.P., Chivers P.T., Parker H.E., Beilin L., Kendall G., Larkin D. (2011). The associations between physical activity, screen time and weight from 6 to 14 yrs: The Raine Study. J. Sci. Med. Sport.

[41] Kantanista A., Osinski W. (2014). Underweight in 14 to 16 year-old girls and boys: Prevalence and associations with physical activity and sedentary activities. Ann. Agric. Environ. Med. Aaem.

[42] Ainsworth B.E., Haskell W.L., Whitt M.C., Irwin M.L., Swartz A.M., Strath S.J., O’Brien W.L., Bassett D.R.J., Schmitz K.H., Emplaincourt P.O. (2000). Compendium of physical activities: An update of activity codes and MET intensities. Med. Sci. Sports Exerc..

